# Isolation and biological activity of triglycerides of the fermented mushroom of *Coprinus Comatus*

**DOI:** 10.1186/1472-6882-12-52

**Published:** 2012-04-24

**Authors:** Jun Ren, Jin-Li Shi, Chun-Chao Han, Zhen-Quan Liu, Jian-You Guo

**Affiliations:** 1 Department of Psychology, Zhejiang Normal University, Jinhua, 321004, P. R. China; 2 School of Pharmacy, Beijing University of Chinese Medicine, Beijing, 100102, P. R. China; 3 School of Pharmacy, Shandong University of Traditional Chinese Medicine, Jinan, 250355, P. R. China; 4 School of Basic Medical Sciences, Beijing University of Chinese Medicine, Beijing, 100029, P. R. China; 5 Key Laboratory of Mental Health, Institute of Psychology, Chinese Academy of Sciences, Beijing, 100101, P. R. China

## Abstract

**Background:**

Although many physiological functions of *Coprinus comatus* have been reported, there has been no report on the antinociceptive activity of *Coprinus comatus*. Therefore, the objective of the present study is to demonstrate the production, isolation, and biological properties of triglycerides (TFC) of the fermented mushroom of *Coprinus comatus.*

**Methods:**

The effects of TFC on cytokines levels, total antioxidant activity, antinociceptive effects *in vivo*, LD_50_ and tactile hyperalgesia were analyzed respectively.

**Results:**

TFC treatment decreased the levels of cytokines and total antioxidant status (TAOS) and inhibited the acetic acid-induced abdominal constrictions in mice. In addition, TFC reduced CFA-induced tactile hyperalgesia in a dose-dependent manner and the LD_50_ of TFC was determined to be 400 mg/kg. However, TFC did not significantly inhibit the reaction time to thermal stimuli in the hot-plate test.

**Conclusions:**

TFC showed anti-inflammatory, antioxidant, peripheral antinociceptive and antihyperalgesic activity in various models of inflammatory pain. The data suggest that TFC may be a viable treatment option for inflammatory pain.

## Background

Edible fungi have a long history of use in Traditional Chinese Medicine [1]. *Coprinus comatus* is a basidiomycete fungus claimed to benefit glycaemic control in diabetes. Many physiological functions of Coprinus comatus have been reported, such as anti-cancer properties [2], immunomodulatory [3] and hypoglycemic activity [4]. Stadler et al. reported that agaricoglycerides, a new class of fungal secondary metabolites isolated from several basidiomycetes, showed strong activities against neurolysin, a protease involved in the regulation of dynorphin and neurotensin metabolism, and even exhibited moderate analgesic *in vivo*[5]. The agaricoglycerides are a new class of fungal secondary metabolites that constitute esters of chlorinated 4-hydroxy benzoic acid and glycerol. Although the crude extract from *Coprinus comatus* was no beneficial effect at lower doses in mice subjected to acetic-acid writhing test, formalin test and hot-plate test [6], there has been no report on the antinociceptive activity of agaricoglycerides extract from *Coprinus comatus.* Therefore, the objectives of the present study were to demonstrate the production, isolation, and biological properties of triglycerides of the fermented mushroom of *Coprinus comatus.*

## Methods

### Fermented mushroom of *Coprinus comatus*

The seed of *Coprinus comatus* was purchased from the Agricultural Culture Collection of China.

First, the seed was grown at 28°C for 5 days on PDA slants (1,000 mL 20% potato extract liquid +20.0 g dextrose +20.0 g agar) and then maintained at 4°C in a refrigerator. Five to six pieces of the mycelia of *Coprinus comatus* were transferred from a slant into 250 mL Erlenmeyer flasks containing 100 mL liquid medium (20% potato extract liquid +2.0% dextrose +0.1% KH_2_PO_4_ + 0.05% MgSO_2_). The culture was incubated at 27°C on a rotary shaker at 180 rpm for 5 days.

A 120-h-old liquid culture was homogenized using a sterilized blender and then inoculated to 500 mL Erlenmeyer flasks containing 300 mL of fermented culture medium. The volume of inoculum was 15 mL, which was then cultivated under the same condition. The 168-h-old culture medium was used for extraction [7-9].

### Triglycerides extracted from fermented mushroom of *Coprinus comatus* (TFC)

The methodology for extraction of triglycerides was described in detail by Han et al. [10]. Briefly, mycelia were separated from the culture fluid by filtration and extracted twice with acetone in an ultrasonic bath. The extract was filtered, and the acetone was removed *in vacuo* to yield an aqueous residue. This residue was diluted with tap water and subsequently extracted three times with EtOAc. The combined organic phases were dried over Na_2_SO_4_ and evaporated *in vacuo* to yield an oily residue.

### Acute toxicity study

When there is no information of TFC on toxicity, it is recommended to use the starting dose of 250 mg/kg body weight. The animals were given intraperitoneally the lowest with dose of 250 mg/kg of the compounds at the first instance. If more than 50% mice die within 24 hrs, another group was chosen at a dose of 200 mg/kg. If no death was observed, increasing dose up to limit dose of 2,000 mg/kg was injected to the mice. If more than 50% death was observed in limit test, the dose was then reduced to 800 mg/kg. Acute half lethal dose (LD_50_) was determined after observing toxic reaction for three days.

### Anti-inflammatory effect of TFC on cytokines levels

Wistar rats (weighing 225 ± 25 g) were used in the study. This study was performed in accordance with the Guide for the Care and Use of Laboratory Animals. The study was approved by the ethics committee of Chinese Academy of Sciences, and all procedures complied with the guidance set out in the Guidelines for Caring for Experimental Animals published by the Ministry of Science and Technology of the People's Republic of China. Care was taken to minimize discomfort, distress, and pain to the animals. Forty healthy female adult rats were randomly divided in four groups. The TFC were presolubilized in mixture of DMSO, ethanol and polysorbate-80. This mixture was subsequently filled up with physiological saline to final concentrations of 4% DMSO, 10% ethanol and 20% polysorbate-80. Intraperitoneal doses of 10, 20 and 30 mg/kg body weight were applied in a volume of 2 ml/kg 1 h before the injection of carrageenan. Diclofenac sodium (1.7 mg/kg) was used as a standard. Inflammatory response was induced by a single intrapleural injection of 0.1 mLof sterile saline solution (NaCl, 0.95%) plus carrageenan (Cg, 1%). Ten healthy rats treated only with intrapleural injection (i.p.) of sterile saline solution (NaCl 0.95%) used as control group. Six hours after the injection of carrageenan, blood samples were drawn from orbital vein from all the groups and serum was separated for biochemical estimations.

The level of TNF-α, IL-1β, VEGF-α, and IL-17 in the rat blood samples were measured as previously described by Cai et al. [11]. Briefly, protein was extracted from the blood samples and the concentration was adjusted to 4 mg/ml. The concentration of TNF-α, IL-1β, VEGF-α, and IL-17 were then determined using a commercial ELISA kit (Shanghai Jinma Biological Technology, Inc., China) following the manufacture’s instruction.

### Measurement of total antioxidant status

The total antioxidant status (TAOS) of serum was determined as previously described by Laight et al. [12]. The increase of absorbance at 405 nm was measured by a microplate reader (Shanghai Xunda Medical Technology, Inc., China).

### Peripheral antinociceptive effect of TFC in animal model

Kunming outbred mice weighing 20-22 g, were purchased from the Experimental Animal Center, Shandong University. The mice were maintained at room temperature under alternating natural light/dark photoperiod, and had access to standard laboratory food and fresh water *ad libitum.*

Writhing test is used for the evaluation of peripheral analgesic activity. Mice were treated (intraperitoneal) with TFC of 10, 20 and 30 mg/kg body weight 60 min before receiving a 0.6% acetic acid injection (10 mL/kg, i.p.). The number of contractions or writhings, determined by abdominal muscle contractions and hind paw extension was recorded for 20 min, starting 10 min after the administration of acetic acid. Diclofenac sodium (1.7 mg/kg) was used as standard.

### Central antinociceptive effect of TFC in animal model

The hot-plate test is commonly used to assess narcotic analgesics or other centrally acting drugs [13,14]. In this test, mice were pre-selected according to their reactions to a thermal stimulus (jumping or licking of hind limbs when placed on a hot plate at 55°C). Latency times were recorded immediately before and 30, 60 and 90 min after drug administration (intrapleural injection with TFC of 10, 20and 30 mg/kg body weight, diclofanac sodium 1.7 mg/kg and sterile saline), up to a maximum time of 40 s to avoid paw lesions [15].

### Anti-hyperalgesia effect of TFC in animal model

Mice received a single intraplantar injection of 100 μl of 1 mg/ml dose of heat-killed and dried Mycobacterium tuberculosis in a mixture of paraffin oil and mannide monoleate. The tactile hyperalgesia was tested as tactile withdrawal threshold before and at 15, 30 and 60 min after drug administration.

### Statistical analysis

The data were expressed as mean ± S.E.M. and results were analyzed by ANOVA followed by Dunnett’s *t* test. P < 0.05 was considered significant.

## Results and discussion

The present study demonstrates, for the first time, that the anti-inflammatory and analgesic effects of TFC. Carrageenan-induced inflammation in rats is a well-characterized experimental model of acute inflammation that permits the quantification and correlation of cellular migration with changes in other inflammatory parameters [16]. Diclofenac is a nonsteroidal anti-inflammatory drug used to treat pain andinflammation associated with arthritis. It was used as a standard. The inflammatory response involves a complex intercellular signal promoting cytokine release. Activated inflammatory cells synthesize and secrete proinflammatory cytokines such as TNF-α, IL-1β, VEGF-α, and IL-17 [17-19]. The suppression of these proinflammatory mediators has been found to reduce the severity of the inflammatory reaction [20]. Some work has been done on the effects of the mushroom on cytokines and nuclear factor kappa B (NF-κB) levels [21-25]. The present study was undertaken to determine the effect of TFC on protein levels of TNF-α, IL-1β, VEGF-α, and IL-17 during the inflammatory process induced by carrageenan.

Treatment with TFC (30 m/kg) significantly reduced the levels of TNF-α by 58.3% (P <0 .01), IL-1β by 27% (P < 0.05),VEG F-α by 46.5% (P < 0.01) and IL-17 by 89.2% (P <0 .001), respectively (Table 1). Results of this study clearly indicate the anti-inflammatory activity of the TFC in acute inflammatory conditions. It could effectively inhibit the leukocyte migration promoted by carrageenan in rat.

**Table 1:** Effect of TFC on the Protein Levels of TNF-α, IL-1β, VEGF-α, and IL-17

Different groups	TNF-α (pg/mL)	IL-1β(pg/mL)	VEGF-α(pg/mL)	IL-17(pg/mL)
Saline group	3021.0 ± 388.4	1066.6 ± 148.8	1212.2 ± 117.7	867.1 ± 58.4
Dic. group	1620.6 ± 118.0^**^	676.7 ± 64.0 ^**^	675.5 ± 22.5 ^**^	91.5 ± 6.5^***^
TFC 10 group	2718.7 ± 370.2	1084.0 ± 84.3	1090.0 ± 44.1	771.7 ± 75.7
TFC 20 group	2013.4 ± 184.7	866.7 ± 70.3	1054.4 ± 44.5	671.1 ± 35.5
TFC 30 group	1260.4 ± 195.5^**^	777.7 ± 66.3 ^*^	648.0 ± 20.2 ^**^	93.5 ± 9.5^***^

Values are shown as means ± SEM. *p < 0.05 vs. Saline group, **p < 0.01 vs. Saline group, ***p < 0.001 vs. Saline group.

**Figure 1:**
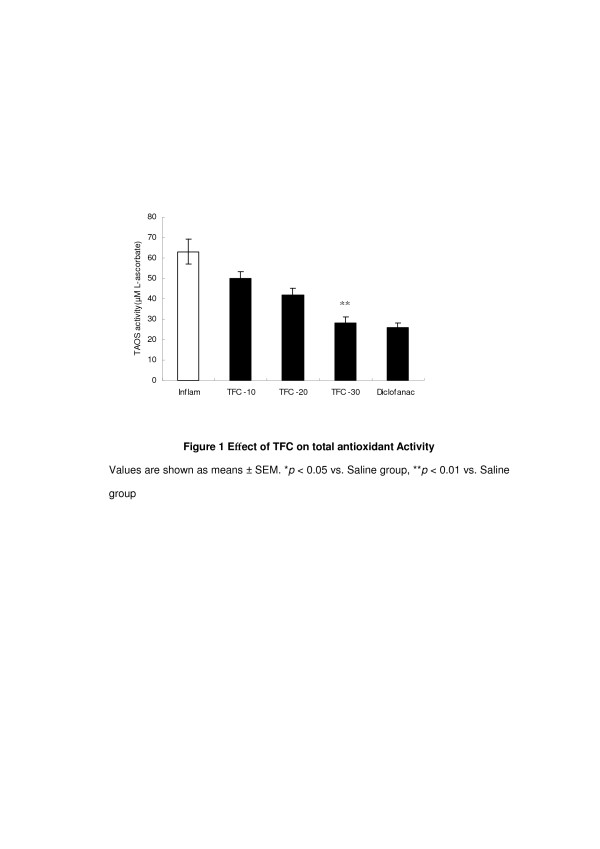
**Effect of TFC on total antioxidant Activity.** Values are shown as means ± SEM. *p < 0.05 vs. Saline group, **p < 0.01 vs. Saline group.

O_2_^−^ has been associated with tissue damage and loss of function during inflammatory episodes [26]. The total antioxidant status (TAOS) is an indication of O_2_^−^ and other oxidant species. We measured TAOS activity as an indirect indication of the formation of O_2_^−^ and other oxidant species. The TFC (10 m/kg) groups had the lower level of TAOS activity in comparison to the saline group (P < 0.01) (Figure 1). It is consistent with the previous report on the antioxidant properties of *Coprinus comatus *[27,28]*.* O_2_^−^ is produced by polymorphonuclear leukocytes and macrophages from the enzyme activity of NADPH oxidase and xanthine oxidase at inflammatory sites. We hypothesized that TFC produce anti-inflammatory effect through decreasing the levels of TAOS activities.

The report that no decrease in oedema induced by formalin injection was observed following treatment with C. comatus also showed that C. comatus had a peripheral antinociceptive and anti-inflammatory effect [29]. In the writhing test, TFC inhibited the acetic acid-induced abdominal constrictions in a dose-dependent manner (40.2%, 69.7%, and 76.9%) (Table 2). The acetic acid-induced writhing reaction in mice has been used as a screening tool for the assessment of analgesic or anti-inflammatory properties of new agents [30]. The constrictions induced by acetic acid in mice result from an acute inflammatory reaction related to the increase in the peritoneal fluid levels of PGE_2_ and PGF_2_α [31]. The fact that TFC was able to inhibit constrictions showed that TFC has a peripheral antinociceptive effect.

**Table 2:** Peripheral antinociceptive effect of TFC in mice subjected to the writhing test

Different groups	Number of contraction (20 min)	Inhibition (%)
Saline group	25.1 ± 2.2	-
Dic. group	5.8 ± 1.9	76.9%
TFC 10 group	15.0 ± 2.1*	40.2%
TFC 20 group	7.6 ± 1.6^**^	69.7%
TFC 30 group	5.8 ± 1.2^**^	76.9%

Values are shown as means ± SEM. *p < 0.05 vs. Distilled water group, **p <0.01 vs. Distilled water group (n = 10).

On the contrary, the result of the hot-plate test did not show that TFC has a central antinociceptive effect. The hot-plate test is commonly used to assess narcotic analgesics or other centrally acting drugs [13,14]. The hot-plate test was performed for the assessment of the central antinociceptive effect of TFC in this study. Results showed that TFC inhibited the reaction time to thermal stimuli at 30, 60, and 90 min compared to controls. However, it was not significant (Table 3). The mechanism of TFC does not exert central antinociceptive effect could be explained that it has poor permeability across the blood brain barrier (BBB). BBB is an active interface between the circulation and the central nervous system (CNS) which restricts the free movement of different substances between the two compartments and plays a crucial role in the maintenance of the homeostasis of the CNS [32].

**Table 3:** Effect of TFC in mice subjected to the hot-plate test. (N = 10)

Groups	Reaction time to the thermal stimulus (s)			
	0 min	30 min	60 min	90 min
Saline group	10.9 ± 0.6	9.6 ± 0.9	8.3 ± 0.80	9.5 ± 0.6
Dic. group	9.9 ± 0.8	13.0 ± 1.1^*^	16.3 ± 0.6**	16.8 ± 1.5**
TFC 10 group	11.9 ± 1.5	11.4 ± 1.5	8.8 ± 1.2	11.4 ± 1.3
TFC 20 group	10.9 ± 2.2	11.5 ± 1.2	9.8 ± 1.1	10.6 ± 1.3
TFC 30 group	13.9 ± 1.1	11.2 ± 0.9	11.4 ± 1.7	12.5 ± 2.9

Values are shown as means ± SD.

*p < 0.05 vs. control group,**p < 0.01 vs. control group.

**Figure 2:**
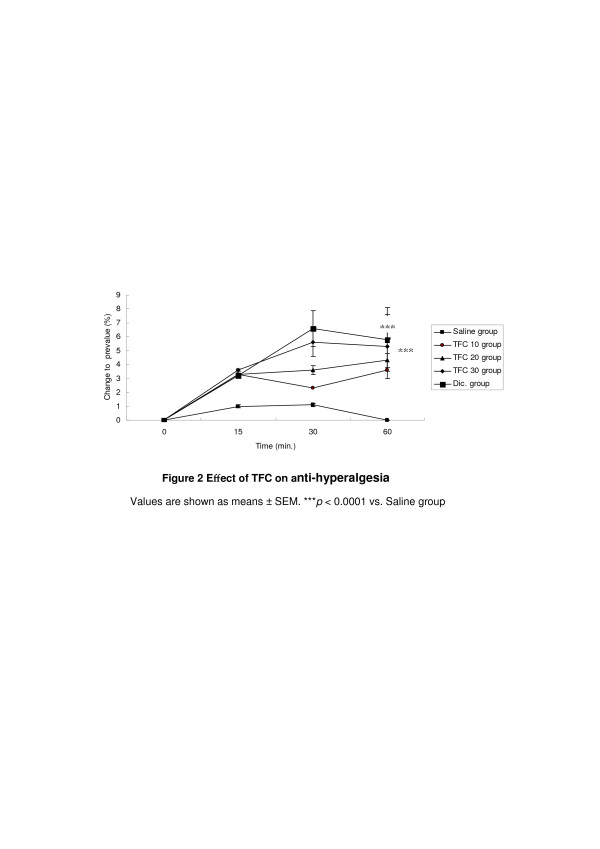
**Effect of TFC on anti-hyperalgesia.** Values are shown as means ± SEM. ***p < 0.0001 vs. Saline group.

Complete Freund’s adjuvant (CFA) -induced hyperalgesia is frequently used as an animal model to study chronic inflammatory pain. The CFA-induced inflammation is accompanied by a tactile hyperalgesia (HA), which is robust over several days [33]. TFC reduced dose-dependently the CFA-induced tactile hyperalgesia (Figure 2). It is likely that the antihyperalgesic effect of CFA was due to a genuine anti-inflammatory effect.

As per the OECD Guidelines-423, LD_50_ was calculated for TFC. When there is no information of TFC on toxicity, it is recommended to use the starting dose of 250 mg/kg body weight. This involves the estimation of the median lethal dose (LD_50_), which is the dose that will kill 50% of the animal population within 72 hours post treatment with the test substance. After intraperitoneal injection, most mice in the 400 mg/kg group were quiet and inactive. Mice died approximately 7 hr after injection. The majority of mice in the 400 mg/kg dose group (5 mice) died within 72 hr following the injection. The LD_50_ was determined to be 400 mg/kg.

## Conclusion

In conclusion, TFC showed anti-inflammatory, antioxidant, peripheral antinociceptive and antihyperalgesic activity in various models of inflammatory pain. The data suggest that TFC may be a viable treatment option for inflammatory pain.

## Competing interests

The authors declare that they have no competing interests.

## Authors’ contributions

J-YG and J-LS were involved in the design of this study and performed laboratory analyses and statistics. JR and C-CH drafted the manuscript along with the other authors. All authors read and approved the final manuscript.

## Pre-publication history

The pre-publication history for this paper can be accessed here:

http://www.biomedcentral.com/1472-6882/12/52/prepub
